# The Anticancer Effect of Fucoidan in PC-3 Prostate Cancer Cells

**DOI:** 10.3390/md11082982

**Published:** 2013-08-19

**Authors:** Hye-Jin Boo, Ji-Young Hong, Sang-Cheol Kim, Jung-Il Kang, Min-Kyoung Kim, Eun-Ji Kim, Jin-Won Hyun, Young-Sang Koh, Eun-Sook Yoo, Jung-Mi Kwon, Hee-Kyoung Kang

**Affiliations:** 1 Department of Pharmacology, School of Medicine, Jeju National University, 102 Jejudaehakno, Jeju 690-756, Korea; E-Mails: wonsein2000@nate.com (H.-J.B.); jyhong7876@hanmail.net (J.-Y.H.); 25008@hanmail.net (S.-C.K.); asdkji@hanmail.net (J.-I.K.); loveis6776@hanmail.net (M.-K.K.); ejk8730@naver.com (E.-J.K.); eunsyoo@jejunu.ac.kr (E.-S.Y.); 2 Department of Biochemistry, School of Medicine, Jeju National University, 102 Jejudaehakno, Jeju 690-756, Korea; E-Mail: jinwonh@jejunu.ac.kr; 3 Department of Microbiology, School of Medicine, Jeju National University, 102 Jejudaehakno, Jeju 690-756, Korea; E-Mail: yskoh7@jejunu.ac.kr; 4 Department of Internal Medicine, School of Medicine, Institute of Medical Sciences, Jeju National University, 102 Jejudaehakno, Jeju 690-756, Korea

**Keywords:** fucoidan, PC-3 cells, apoptosis, Wnt/β-catenin

## Abstract

Fucoidan, a sulfated polysaccharide, has a variety of biological activities, such as anti-cancer, anti-angiogenic and anti-inflammatory. However, the mechanisms of action of fucoidan as an anti-cancer agent have not been fully elucidated. The present study examined the anti-cancer effect of fucoidan obtained from *Undaria pinnatifida* in PC-3 cells, human prostate cancer cells. Fucoidan induced the apoptosis of PC-3 cells by activating both intrinsic and extrinsic pathways. The induction of apoptosis was accompanied by the activation of extracellular signal-regulated kinase mitogen-activated protein kinase (ERK1/2 MAPK) and the inactivation of p38 MAPK and phosphatidylinositol 3-kinase (PI3K)/Akt. In addition, fucoidan also induced the up-regulation of p21^Cip1/Waf^ and down-regulation of E2F-1 cell-cycle-related proteins. Furthermore, in the Wnt/β-catenin pathway, fucoidan activated GSK-3β that resulted in the decrease of β-catenin level, followed by the decrease of c-myc and cyclin D1 expressions, target genes of β-catenin in PC-3 cells. These results suggested that fucoidan treatment could induce intrinsic and extrinsic apoptosis pathways via the activation of ERK1/2 MAPK, the inactivation of p38 MAPK and PI3K/Akt signaling pathway, and the down-regulation of Wnt/β-catenin signaling pathway in PC-3 prostate cancer cells. These data support that fucoidan might have potential for the treatment of prostate cancer.

## 1. Introduction

Fucoidan is a sulfated polysaccharide found in the cell wall matrix of brown seaweed, such as *Ascophyllum nodosum*, *Cladosiphon okamuranus*, *Ecklonia kurome*, *Fucus evanescens*, *Fucus vesiculosus*, *Hizikia fusiforme*, *Laminaria angustata* and *Undaria pinnatifida* [1,2,3]. Structurally, fucoidan is a heparin-like molecule with a substantial percentage of l-fucose, sulfated ester groups, as well as small proportions of d-xylose, d-galactose, d-mannose, and glucuronic acid [4]. Among the several kinds of fucoidans, the main one is a sulfated polysaccharide of fucodian from *Undaria pinnatifida*, described as sulfated galactofucan [5]. Fucoidan has various biological activities, such as anti-cancer [6], anti-inflammatory, anti-angiogenic [7], anti-coagulant [8] and anti-HIV [9] activities. However, the action mechanism of fucoidan as an anti-cancer agent has not been fully elucidated.

Prostate cancer is the most commonly diagnosed cancer and second leading cause of mortality in males in industrialized countries [10]. The incidence of prostate cancer in Asian countries is lower than that in Western countries; however, the mortality of prostate cancer is increasing rapidly in Asian males due to westernization of dietary life style [11]. Early-stage prostate cancer requires androgens for growth but eventually regresses to an androgen-independent stage, and progresses despite androgen ablation [12]. The molecular mechanisms for androgen-independent cancer progression are poorly understood. Among prostate cancer cell lines, the PC-3 cell line is known to be analogous to androgen-independent cancer cells [13].

The up-regulation of the Wnt/β-catenin pathway has been found in a large portion of prostate cancer patients in several reports [14]. The Wnt ligands that belong to a family of secreted cysteine-rich glycoproteins, have been described to play various roles during early development and tumorigenesis [15]. The increased expression of β-catenin, a key component of the canonical Wnt signaling pathway, plays a pivotal role in many cancers. The level of free β-catenin is strongly regulated by a β-catenin degradation complex. In the absence of a Wnt signal, the β-catenin level is constitutively decreased by a β-catenin degradation complex, including axin, adenomatous polyposis coli (APC), casein kinase I and GSK-3β [16]. In the presence of the Wnt signal, the degradation of β-catenin is prevented, which results in the accumulation of β-catenin in the nucleus, a characteristic of the Wnt signaling pathway activation. The nuclear accumulation of β-catenin promotes transcriptional activity of lymphoid enhancer-binding factor (LEF)/T-cell factor (TCF) transcription factors in the nucleus, thereby Wnt target genes (c-myc, cyclin D1, and MMP-7, *etc*.) are activated. Among the proteins of the β-catenin destruction complex, GSK-3β plays a pivotal role in the Wnt pathway. GSK-3β is also prevented via Wnt signaling, which may contribute to the progression of prostate cancer [17].

The present study demonstrates the anticancer effect of fucoidan on apoptosis induction by the down-regulation of the Wnt/β-catenin pathway in PC-3 human prostate cancer cells.

## 2. Results

### 2.1. Inhibitory Effect of Fucoidan on the Growth of PC-3 Cells

**Figure 1:**
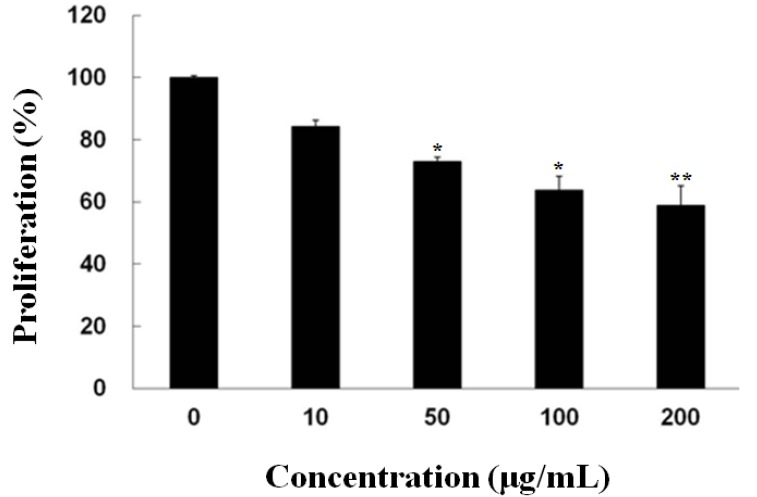
Fucoidan inhibited the proliferation of PC-3 cells. Cell proliferation inhibition was measured by methylthiazoletetrazolium (MTT) assay. Data are presented as mean ± SD from three independent experiments. * *p* < 0.05, ** *p* < 0.01, compared with the control (control; without fucoidan).

To examine the effect of fucoidan on the growth of PC-3 cells, cell viability was evaluated using the methylthiazoletetrazolium (MTT) assay. Fucoidan treatment induced dose-dependent cell death (10 μg/mL, 15.2%; 50 μg/mL, 29.8%; 100 μg/mL, 39.3%; 200 μg/mL, 45.1%) (Figure 1). These results indicate that fucoidan could inhibit the growth of PC-3 cells in a dose-dependent manner.

### 2.2. Fucoidan Induced Apoptotic Characteristics in PC-3 Cells

**Figure 2:**
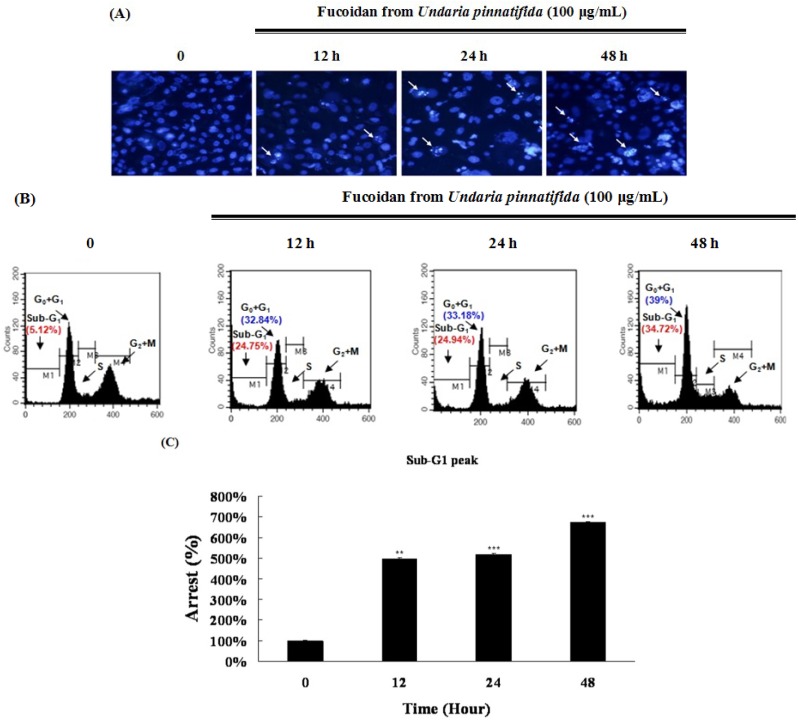
Fucoidan led to apoptotic characteristics in PC-3 cells. (**A**) PC-3 cells were stained with DNA-specific fluorescent dye, Hoechst 33342. Apoptotic bodies were observed by an inverted fluorescent microscope equipped with an IX-71 Olympus camera (magnification ×200); (**B**) The cell cycle analysis was performed by flow cytometry; (**C**) The cell percentage of sub-G_1_ peak in the cell cycle. Data are presented as mean ± SD from three independent experiments. * *p* < 0.05, ** *p* < 0.01, and *** *p* < 0.001 compared with the control (control; without fucoidan).

We investigated whether the inhibitory effect of fucoidan on the growth of the PC-3 cells resulted from apoptosis induction. The morphological changes in the nucleus and all the crucial biochemical parameters of apoptosis induced by fucoidan were examined. Apoptotic bodies were observed by Hoechst 33342 staining in fucoidan-treated cells, but not in fucoidan non-treated cells (Figure 2A). This result indicates that fucoidan can be effective in the induction of apoptotic morphological changes, such as chromatin condensation, membrane blebbing and cell shrinkage. In order to evaluate the effect of fucoidan on the increase of the hypodiploid cell proportion, a cell cycle analysis was performed by propidium iodide (PI) staining. Figure 2B,C show that the percentage of sub-G_1_ fraction increases after stimulation with 100 μg/mL of fucoidan with treatments at various points in time (12 h, 24.75%; 24 h, 24.94%; 48 h, 34.72%). These results show that fucoidan could induce apoptosis of the PC-3 cells. 

### 2.3. Fucoidan Induced Apoptosis through Extrinsic and Intrinsic Apoptosis Pathways in PC-3 Cells

**Figure 3:**
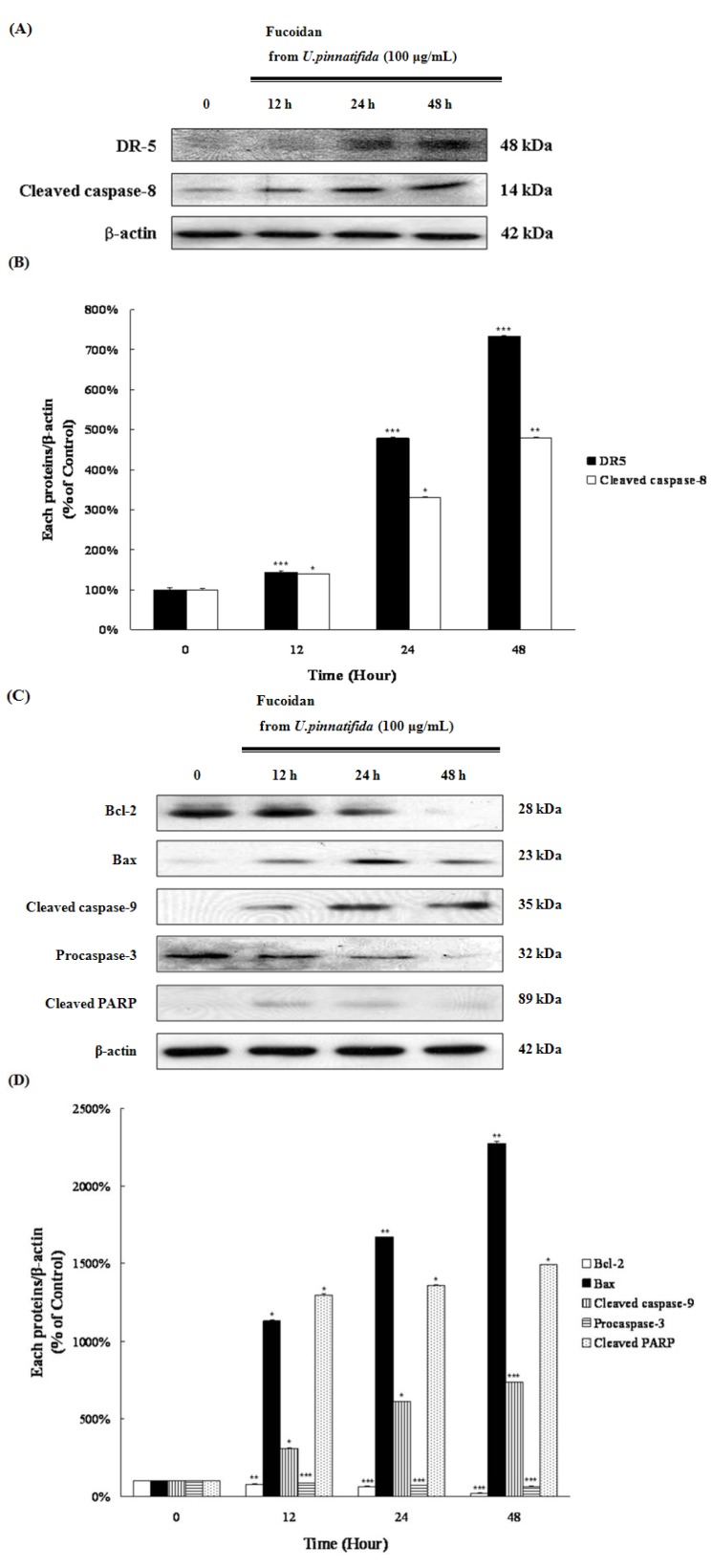
Effect of fucoidan on the levels of extrinsic and intrinsic apoptosis pathways-related proteins. (**A**) DR-5 and cleaved caspase-8 levels were examined by Western blot; (**B**) Data represent the percentage of DR5 and cleaved caspase-8 expressions in PC-3 cells; (**C**) Lysates were analyzed for the expression of Bcl-2, Bax, cleaved caspase-9, procaspase-3 and cleaved PARP by Western blot; (**D**) Data represent the percentage of Bcl-2, Bax, cleaved caspase-9, procaspase-3 and cleaved PARP expressions in PC-3 cells. Data are presented as mean ± SD from three independent experiments. * *p* < 0.01, ** *p* < 0.05, and *** *p* < 0.001 compared with the control (control; without fucoidan).

Apoptotic cell death results from extrinsic and intrinsic molecular signaling pathways [18]. Fucoidan treatment induced the activation of extrinsic pathway-related proteins, DR5 and caspase-8, as well as the activation of the intrinsic pathway through the decrease of Bcl-2, the increase of Bax, and the activation of caspase-9, which were followed by the activation of caspase-3 and the cleavage of poly(ADP-ribose)-polymerase (PARP) (Figure 3A-D).

### 2.4. Effect of Fucoidan on MAP Kinase and PI3K/Akt Signaling in PC-3 Cells

**Figure 4:**
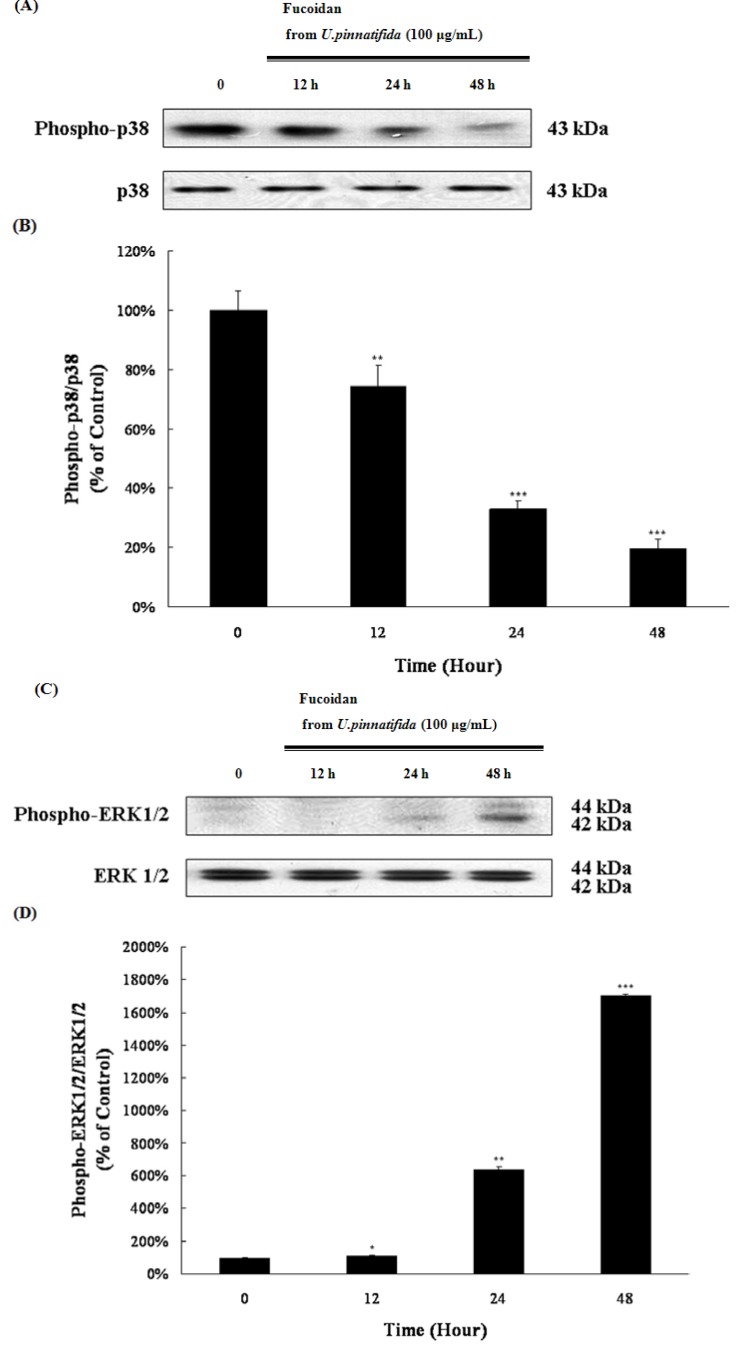
Effect of fucoidan on mitogen-activated protein (MAP) kinase signaling. The levels of phospho-p38 and p38 (**A**) as well as phospho-ERK1/2 and ERK1/2 (**C**) were examined by Western blot. Data represent the percentage of phospho-p38 and p38 (**B**) as well as phospho-ERK1/2 and ERK1/2 (**D**) levels in PC-3 cells. Data are presented as mean ± SD from three independent experiments. * *p* < 0.01, ** *p* < 0.05, and *** *p* < 0.001 compared with the control (control; without fucoidan).

**Figure 5:**
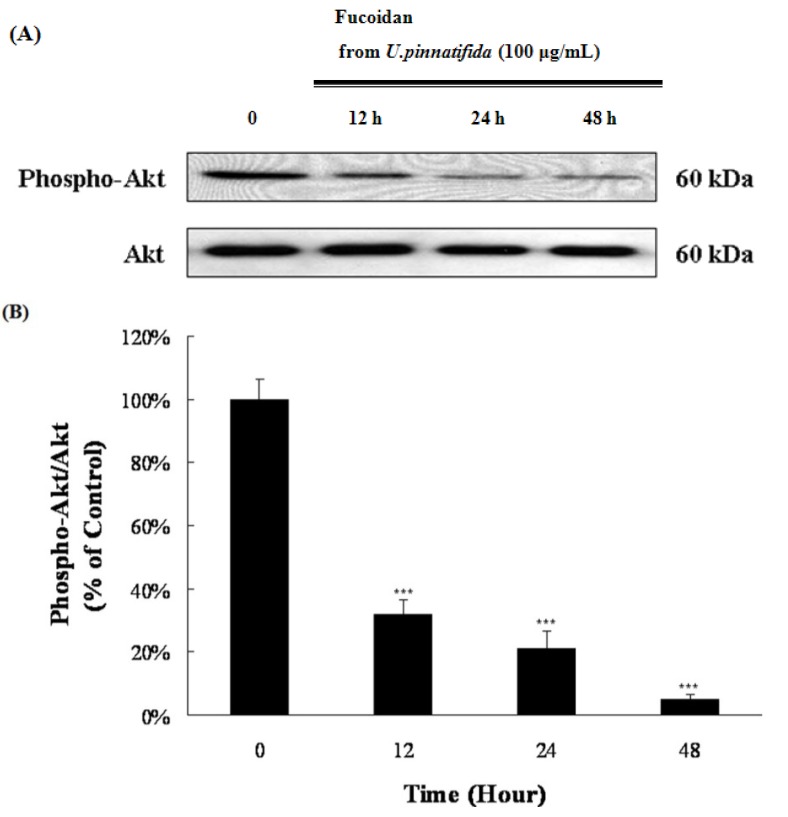
Effect of fucoidan on PI3K/Akt signaling. (**A**) Lysates were analyzed for the levels of phospho-Akt and Akt by Western blot; (**B**) Data represent the percentage of phospho-Akt level in PC-3 cells. Data are presented as mean ± SD from three independent experiments. * *p* < 0.05, ** *p* < 0.01, and *** *p* < 0.001 compared with the control (control; without fucoidan).

Mitogen-activated protein kinase (MAPK) pathways regulate differentiation, mitosis, proliferation, and apoptosis [19]. In order to establish the MAP kinase mechanism of apoptosis induced by fucoidan, the activation of extracellular signal-regulated kinase (ERK1/2) MAPK and p38 MAPK, following fucoidan treatment, was examined. Fucoidan treatment increased the phospho-ERK1/2 level, whereas the phospho-p38 level decreased (Figure 4A–D). The phosphatidylinositol 3-kinase (PI3K)/Akt signaling pathway also regulates cell survival, cell growth and apoptosis [20]. The activation of PI3K/Akt promotes the proliferation and survival of cancer cells [21]. Fucoidan decreased the phosphor-form of PI3K/Akt (Figure 5A,B). These results suggest that fucoidan might induce apoptosis via the inactivation of the PI3K/Akt pathway and the p38 MAPK pathway, as well as the activation of the ERK1/2 MAPK pathway.

### 2.5. Fucoidan Induced G_0_/G_1_ Phase Arrest of PC-3 Cells

**Figure 6:**
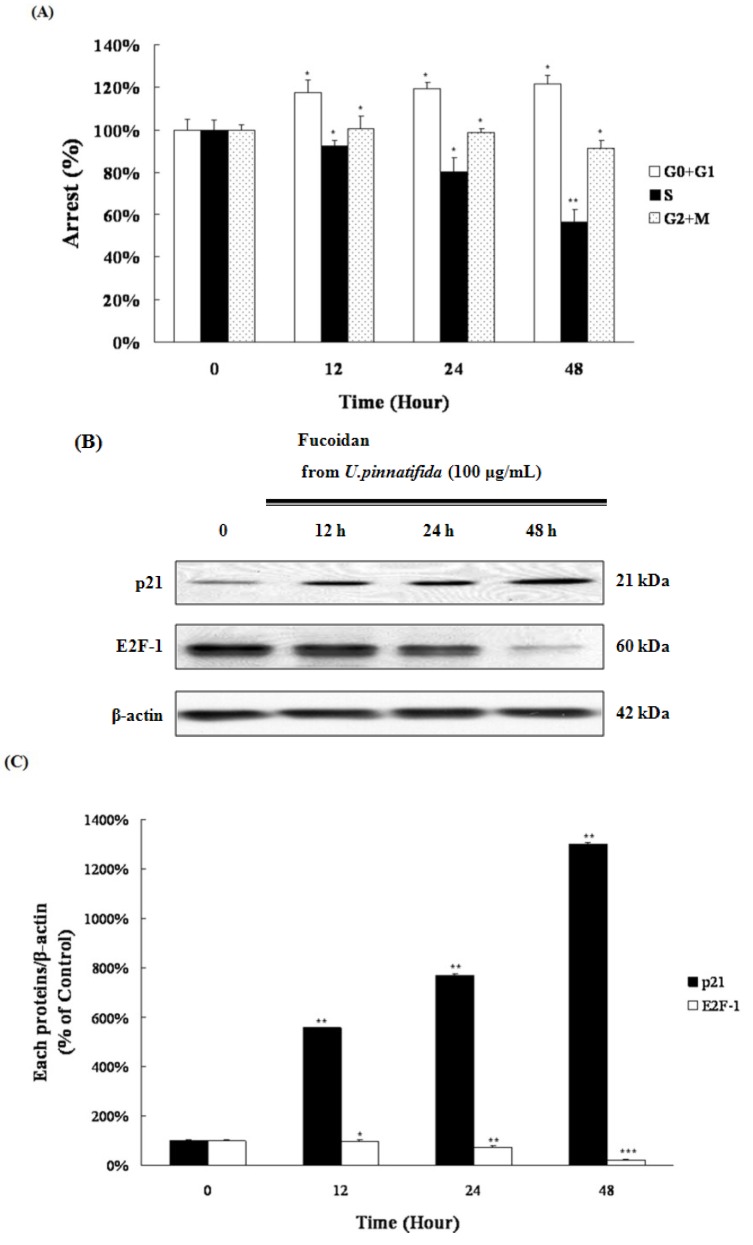
Fucoidan induced arrest of G_0_/G_1_ phase in PC-3 cells. (**A**) Cell cycle analysis was performed by flow cytometry. Data represent the percentage of cells in each phase of the cell cycle; (**B**) The levels of p21 and E2F-1 were examined by Western blot; (**C**) Data represent the percentage of p21 and E2F-1 levels in PC-3 cells. Data are presented as mean ± SD from three independent experiments. * *p* < 0.05, ** *p* < 0.01, and *** *p* < 0.001 compared with the control (control; without fucoidan).

Figure 2B shows that the cell percentage of the G_0_/G_1_ fraction increases at 100 μg/mL of fucoidan in a time-dependent manner (12 h, 32.84%; 24 h, 33.18%; 48 h, 39.0%). These data show that fucoidan could induce arrest of the G_0_/G_1_ phase of the PC-3 cells (Figure 6A).

E2F-1 is an important transcription factor for cell cycle progression from the G_1_ to S phase and DNA synthesis [22]; p21^Cip1/Waf^ is known to regulate the entry of cells at the G_1_-S-Phase transition checkpoint and induce apoptosis [23]. The effect of fucoidan on the level of E2F-1, and p21^Cip1/Waf^ was examined. Fucoidan treatment caused a significant reduction in the expression of E2F-1, whereas the expression of p21 increased significantly (Figure 6B,C). These results indicate that down-regulation of E2F-1 and up-regulation of p21 by fucoidan might contribute the arrest of of G_0_/G_1_ phase of the PC-3 cells. 

### 2.6. Fucoidan Induced Apoptosis via Down-Regulation of Wnt/β-Catenin Pathway in PC-3 Cells

**Figure 7:**
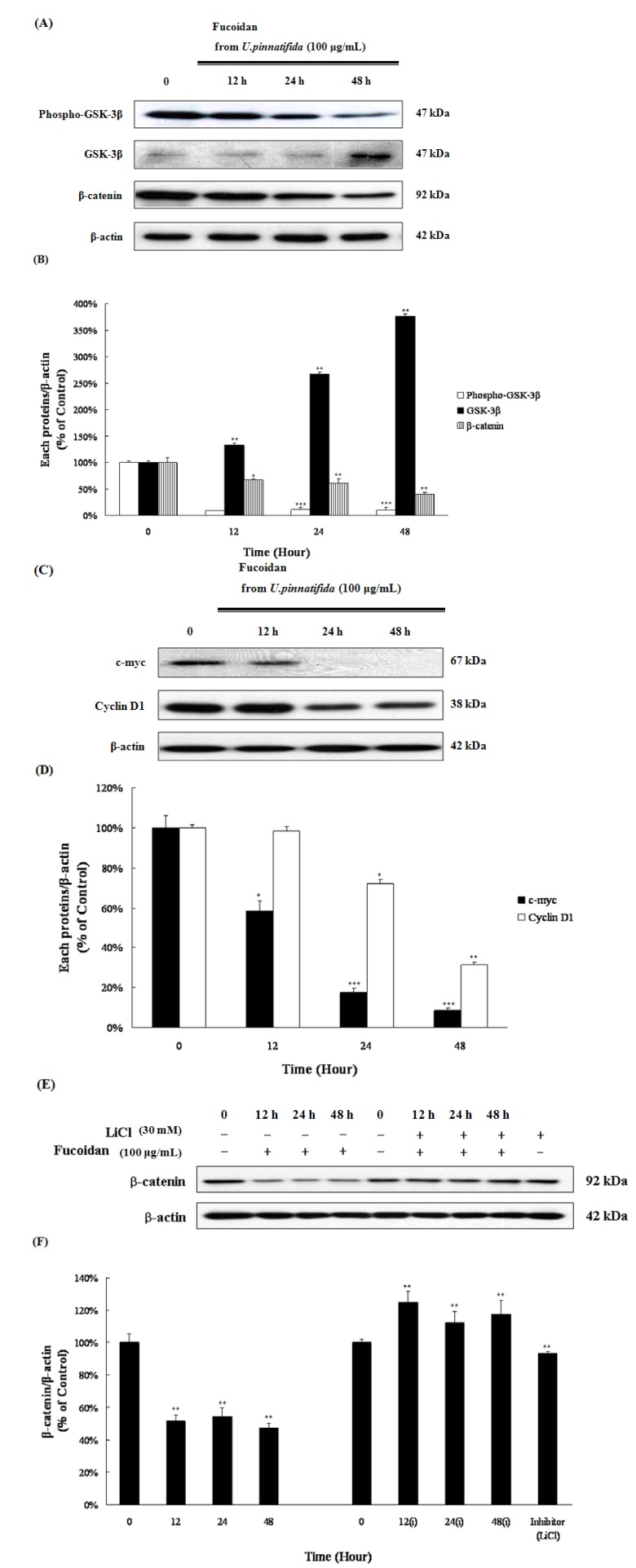
Effect of fucoidan on the levels of the Wnt/β-catenin signaling pathway-related proteins in PC-3 cells. Lysates were analyzed for the levels of phospho-GSK-3β, GSK-3β and β-catenin (**A**) as well as c-myc and cyclin D1 (**C**) by Western blot. Data represent the percentage of phospho-GSK-3β, GSK-3β and β-catenin (**B**) as well as c-myc and cyclin D1 levels (**D**) in PC-3 cells; (**E**) PC-3 cells (1 × 10^5^ cells/mL) were pretreated with 30 mM of GSK-3β inhibitor (LiCl) for 1 h and then treated with 100 μg/mL of fucoidan for 0, 12, 24 and 48 h. The expression of β-catenin was examined by Western blot; (**F**) Data represent the percentage of β-catenin expression in PC-3 cells. Data are presented as mean ± SD from three independent experiments. * *p*< 0.05, ** *p* < 0.01, and *** *p*< 0.001 compared with the control (control; without fucoidan).

An up-regulation of the Wnt/β-catenin signaling pathway has a pivotal role in the development and progression of prostate cancer [24]. After fucoidan treatment, the expression of Wnt/β-catenin signaling-related proteins was examined. As shown Figure 7A,B, fucoidan led to a decrease of β-catenin, a key molecule of Wnt/β-catenin signaling pathway. Fucoidan also induced the activation of GSK-3β, a regulator of β-catenin, which was followed by the decrease of c-myc and cyclin D1, β-catenin target genes (Figure 7C,D). To confirm the role of the Wnt/β-catenin signaling pathway in fucoidan-induced apoptosis, GSK-3β, an important upstream regulator of β-catenin, was inhibited using the GSK-3β inhibitor LiCl. Interestingly, following pretreatment with LiCl, fucoidan restored β-catenin levels to untreated control levels (Figure 7E,F). These data suggest that fucoidan might regulate β-catenin through GSK-3β activation, and that the fucoidan effect on the apoptosis induction could be associated with the down-regulation of the Wnt/β-catenin signaling pathway.

## 3. Discussion

In the present study, it was observed that fucoidan treatment could induce the apoptosis of PC-3 human prostate cancer cells via the activation of the ERK1/2 MAPK signaling pathway, the inactivation of p38 MAPK and PI3K/Akt, and inhibition of the Wnt/β-catenin signaling pathway. 

Previous studies have also indicated that fucoidan from several brown algae directly inhibite the proliferation of various cancer cells, including HS-Sultan [6], U937 [25], MCF-7 [2], HCT-15 [26] and A549 [27] and SMMC-7721 [28] cells in a specific manner without cytotoxicity against the normal cells, such as RAW 264.7 [29] and HEL-299 [26] cells. Although no comparative analysis by an experiment was done, these reports suggest that the anti-cancer effects of fucoidan may have nothing to do with its sources. Indeed, this study confirms that fucoidan could directly inhibit the proliferation of PC-3 cells in a dose-dependent manner (Figure 1). 

Apoptosis is a highly regulated physiologic mechanism of cell death during homeostasis, disease and development [30]; morphologically characterized by chromatin condensation, membrane blebbing, cell shrinkage, and an increased population of sub-G_1_ hypodiploid cells [31]. Fucoidan induced the nuclear morphologic changes of PC-3 cells in line with the physiologic apoptotic process (Figure 2A); treatment with fucoidan (100 μg/mL; approximately 0.75 μM) also increased the sub-G_1_ fraction by 34.72% at 48 h (Figure 2B,C) compared with the control. On the other hand, resveratrol is known to induce apoptosis of prostate cancer cells. When PC-3 cells were treated with resveratrol (25 μM for 96 h), the sub-G_1_ hypodiploid cell population increased by 4.3% as compared to that of the control [32]. Compared with resveratrol, fucoidan appears to be more effective in the induction of apoptosis in the PC-3 cells.

Two key molecular signaling pathways are implicated in the induction of apoptotic cell death. The one is the extrinsic pathway, which is activated by a death receptor from outside the cell; the other is the intrinsic pathway, which is activated by a Bcl-2 protein family and downstream mitochondrial signals from inside the cell [18]. Fucoidan treatment led to the activation of DR5 and cleavage of caspase-8, which are critical in the extrinsic pathway; fucoidan also led to the down-regulation of Bcl-2, up-regulation of Bax, and activation of caspase-9, which are essential in intrinsic pathway (Figure 3A–D). Extrinsic and intrinsic apoptosis pathways induce apoptosis via interaction of MAPK and PI3K/Akt signaling pathways; in other words, these pathways affect each other.

MAPK pathways are known to regulate apoptosis [33]. Among MAPK proteins, ERK1/2 MAPK is known to promote differentiation, survival and proliferation of cells [34], but several reports have indicated that the activation of ERK1/2 MAPK can induce apoptosis [35]. Cisplatin is known to induce apoptosis in the HeLa cells via activation of the ERK pathway [36]. p38 MAPK is known to be activated by stress to modulate cell differentiation, cell cycle, cell growth, inflammation, and cell death [37]; whereas some reports have suggested that p38 MAPK can promote cancer cell growth and survival. Docosahexaenoic acid is reported to induce apoptosis of the A549 cells by down-regulation of p38 MAPK [38]. This study confirmed that fucoidan treatment could activate ERK1/2 MAPK, whereas p38 MAPK was inactivated (Figure 4A–D). Fucoidan treatment decreased the phosphorylation of Akt as expected (Figure 5A,B). Phosphorylation of Akt is reported to be regulated by p38; the results in the present study suggest that fucoidan treatment could inactivate p38 MAPK signaling pathway, followed by the inactivation of PI3K/Akt signaling pathway.

Fucoidan increased the cell fraction of the G_0_/G_1_ phase, whereas the cell percentage of the S phase was decreased (Figure 6A). Among the cell-cycle-related proteins, E2F-1 and p21^Cip1/Waf^ are known to play an important role in the cell cycle progression from the G_1_ to S phase. Fucoidan decreased expression of E2F-1 and increased expression of p21, followed by the inhibition of proceeding from G_1_ to S phase in the PC-3 cells (Figure 6B,C). E2F-1 and p21 are regulated by β-catenin, an essential component of Wnt/β-catenin pathway. 

Wnt/β-catenin signaling plays a pivotal role in the development and progression of prostate cancer. Furthermore, previous a study has suggested that highly invasive androgen-independent prostate cancer cell lines, such as PC-3 and DU-145, display higher levels of Wnt/β-catenin signaling compared with the androgen-dependent prostate cancer cell line, LNCaP, and non-cancerous PWR-1E and PZ-HPV-7 prostate cells [23]. The activation of β-catenin is inhibited by GSK-3β; GSK-3β promotes the phosphorylation on the serine and threonine residues in the amino-terminal region of β-catenin, and thereby targets it for ubiquitination and degradation via the ubiquitin proteasome pathway by βTrCP or Siah [16]. In the present study, fucoidan treatment was able decrease β-catenin levels through the activation of GSK-3β (Figure 7A,B). The activity of GSK-3β is diminished through the phosphorylation of serine 9, which is known to be done by Akt [17]. Fucoidan treatment decreased phospho-Akt level, followed by the inhibition of GSK-3β phosphorylation (Figure 5A,B). These results suggest that fucoidan could decrease β-catenin level through the inactivation of Akt and the activation of GSK-3β. When pretreated with LiCl, a GSK-3β inhibitor, and then treated with fucoidan, the level of β-catenin was restored to vehicle-treated level. The results support the hypothesis that fucoidan could regulate the level of β-catenin via the Wnt/β-catenin signaling pathway (Figure 7E,F). Fucoidan treatment led to a down-regulation of c-myc and cyclin D1, which are known to be β-catenin target proteins (Figure 7C,D). These results might demonstrate that induction of apoptosis and cell cycle arrest by fucoidan were accompanied by a down-regulation of the Wnt/β-catenin signaling pathway.

Fucoidan is likely to be administered parenterally, because of its high-molecular weight. In recent reports, intraperitoneal administration of fucoidan was able to inhibit breast cancer cell growth in a mouse model [39,40]. On the other hand, effects of fucoidan are known to be mediated by class A and class B scavenger receptors (SR-A and SR-B) [41,42,43], and prostate cancer cells including PC-3 express SR-B1 [44,45]. Therefore, fucoidan may have therapeutic potential for prostate cancer treatment.

## 4. Experimental Section

### 4.1. Materials

Fucoidan (from *Undaria pinnatifida*) was purchased from Sigma-Aldrich Korea (Sigma-Aldrich Korea, Kyunggi-do, Korea), 3-(4,5-dimethylthiazol-2-yl)-2,5-diphenyltetrazolium bromide (MTT), Hoechst 33342, propidium iodide (PI) and lithium chloride (LiCl) were purchased from Sigma (Sigma Chemical Co., St. Louis, MO, USA). The anti-Bcl-2, anti-c-myc, anti-Bax, anti-procaspase-3, anti-β-catenin, anti-E2F-1, anti-DR5, anti-GSK-3β and anti-phospho-GSK-3β were purchased from Santa Cruz Biotechnology (Santa Cruz Biotech, Paso Robles, CA, USA); anti-p38, anti-phospho-p38, anti-Akt, anti-phospho-Akt, anti-ERK1/2, anti-phospho-ERK1/2, anti-cleaved poly(ADP-ribose)polymerase (PARP) and anti-cleaved caspase-9 were purchased from Cell Signaling Technology (Cell Signaling Technology, Beverly, MA, USA); anti-cleaved caspase-8, anti-p21 and cyclin D1 were purchased from BD Biosciences (BD Biosciences, San Diego, CA, USA).

### 4.2. Cell Culture

PC-3, a human prostate cancer cell line, was obtained from the Korean Cell Line Bank (KCLB) and cultured in Roswell Park Memorial Institute medium (RPMI) 1640 (Hyclone, Logan, UT, USA) supplemented with 10% fetal bovine serum (Hyclone, UT, USA), 100 U/mL penicillin and 100 mg/mL streptomycin (GIBCO Inc., Grand Island, NY, USA) at 37 °C in a humidified atmosphere with 5% CO_2_.

### 4.3. Cell Viability Assay

The effect of fucoidan on the growth of PC-3 cells was evaluated using the MTT assay [46]. The cells (1 × 10^5^ cells/mL) were seeded in 200 μL on 96-well microplates. After 18 h incubation to allow cell attachment, the cells were treated with fucoidan (10, 50, 100 and 200 μg/mL) for 72 h. The cells were treated with 50 μL (5 mg/mL) MTT dye and incubated 37 °C for 4 h. The medium was aspirated and 150 μL/well dimethyl sulfoxide was added to dissolve the formazan precipitate. Cell viabilities were determined by measuring the absorbance at 540 nm using a microplate enzyme-linked immunosorbent assay (ELISA) reader (BioTek Instruments, Inc., Winooski, VT, USA). Each experiment was repeated at least three times.

### 4.4. Flow Cytometric Analysis of Apoptosis

The effect of fucoidan on cell cycle distribution was analyzed by flow cytometry after staining the cells with PI [47]. PC-3 cells (1 × 10^5^ cells/mL) were treated with 100 μg/mL of fucoidan and cultured for 12, 24 and 48 h. The treated cells were trypsinized, washed twice with phosphate-buffered saline (PBS) and fixed with 70% ethanol for 30 min at −20 °C. The fixed cells were washed twice with cold PBS, incubated with 50 μg/mL RNase A at 37 °C for 30 min, and stained with 50 μg/mL PI in the dark for 30 min at 37 °C. The stained cells were analyzed using fluorescence activated cell sorter (FACS) caliber flow cytometry (Becton Dickinson, San Jose, CA, USA). The proportion of cells in G_0_/G_1_, S and G_2_/M phases was represented as DNA histograms. Apoptotic cells with hypodiploid DNA were measured by quantifying the sub-G_1_ peak in the cell cycle pattern. For each experiment, 10,000 events per sample were analyzed, and experiments were repeated three times.

### 4.5. Morphological Analysis of Apoptosis by Hoechst 33342 Staining

For the detection of apoptosis, the PC-3 cells were seeded at 1 × 10^5^ cells/mL on 24-well microplates. After 18 h incubation to allow cell attachment, the cells were treated with 100 μg/mL of fucoidan and cultured for 12, 24 and 48 h. The cells were incubated in a Hoechst 33342 staining solution in a final concentration of 10 μg/mL at 37 °C for 20 min. The stained cells were observed with an inverted fluorescent microscope equipped with an IX-71 Olympus camera and photographed (magnification ×200).

### 4.6. Western Blot Analyses

The PC-3 cells were treated with 100 μg/mL of fucoidan and incubated for 12, 24 and 48 h. The cells were harvested and washed twice with cold PBS. The cells were lysed with lysis buffer (50 mM Tris-HCl (pH 7.5), 150 mM NaCl, 2 mM EDTA, 1 mM EGTA, 1 mM NaVO_3_, 10 mM NaF, 1 mM dithiothreitol (DTT), 1 mM phenylmethylsulfonylfluoride (PMSF), 25 μg/mL aprotinin, 25 μg/mL leupeptin, and 1% nonidet P-40) and kept on ice for 30 min at 4 °C. The lysates were centrifuged at 15,000 rpm at 4 °C for 15 min. The supernatants were stored at −20 °C until use. Protein content was determined by Bradford assay [48]. The same amount of lysates were separated on 6%–10% sodium dodecyl sulfate polyacrylamide gel electrophoresis (SDS-PAGE) gels and then transferred onto a polyvinylidene fluoride (PVDF) membrane (BIO-RAD, Hercules, CA, USA) by glycine transfer buffer (192 mM glycine, 25 mM Tris-HCl (pH 8.8), and 20% MeOH (v/v)) at 150 V for 90 min. After blocking with 5% nonfat dried milk, the membrane was incubated with primary antibody and then with a secondary horseradish peroxidase (HRP) antibody (1:10,000) at room temperature. The membrane was exposed on X-ray films (Agfa-Gaevert, Antwerp, Belgium), and protein bands were detected using a WEST-ZOL^®^ plus Western Blot Detection System (iNtRON, Gyeonggi-do, Korea). An anti-β-actin (Sigma Chemical Co., St. Louis, MO, USA) was used as a loading control.

### 4.7. Statistical Analyses

All results were expressed as the means ± standard deviation (SD) of at least three independent experiments. Student’s *t*-test was used to evaluate the data with the following significance levels: * *p* < 0.05, ** *p* < 0.01 and *** *p* < 0.001.

## 5. Conclusions

In conclusion, fucoidan obtained from *Undaria pinnatifida* induced intrinsic and extrinsic apoptosis pathways through the activation of the ERK1/2 MAPK, and the inactivation of the p38 MAPK and PI3K/Akt signaling pathway. Moreover, the induction of apoptosis by fucoidan was accompanied by a down-regulation of the Wnt/β-catenin signaling pathway. The results demonstrate that fucoidan might have therapeutic potential for prostate cancer treatment. 
